# Maternal and child social support and food availability in relation to child growth in four low- and middle-income countries

**DOI:** 10.1038/s41598-022-09850-1

**Published:** 2022-04-08

**Authors:** Hwa-Young Lee, In Han Song, Ichiro Kawachi

**Affiliations:** 1 Department of Global Health and Population, Harvard T.H. Chan School of Public Health, 677 Huntington Ave, Boston, MA 20115 USA; 2 Institute of Convergence Science (ICONS), Convergence Science Academy, Yonsei University, 50 Yonsei-ro Seodaemun-gu, Seoul, 03722 Republic of Korea; 3 Graduate School of Social Welfare, Yonsei University, 50 Yonsei-ro Seodaemun-gu, Seoul, 03722 Republic of Korea; 4 Department of Social and Behavioral Sciences, Harvard T.H. Chan School of Public Health, 677 Huntington Ave, Boston, MA 20115 USA

**Keywords:** Health policy, Nutrition, Paediatrics

## Abstract

Previous studies showed positive associations between specific types of social capital and child nutritional status. Our study examined whether improved food availability mediates the impact of maternal and child social support on child nutritional status in four low- and middle-income countries. We used data from the Young Lives cohort study, comprising 1,000 children aged 8 and 12 in Vietnam and Ethiopia, 1008 in India, and 714 in Peru. The outcome variables were the z-scores for height for age and body mass index (HAZ and BAZ, respectively). The causal mediation analysis framework was used. In Peru, above-median values of maternal social support and receiving child financial support were positively associated with HAZ at age 12. The level of maternal financial support was positively associated with BAZ among 12-year-old children in India. Peru was the only country where a positive association was found between food availability and maternal financial support among children aged 12. However, food availability did not mediate the effect of maternal financial support on HAZ at age 12. Strengthening social support to improve child nutritional status, especially by improving food availability, may not be a sufficient intervention in resource-poor settings because sources of support may lack sufficient food resources to share. However, more comprehensive measurements of social support and food security are necessary to better understand the mechanism of social support and child nutritional status.

## Introduction

Poor nutritional status in childhood has been linked to an elevated risk of mortality and morbidity later in life^1^. Not only does impaired physical growth hamper child development (defined as the attainment of gross motor and fine motor skills), psychosocial competencies, and cognitive abilities^2,3^, it also raises the risk of contracting infectious diseases^4^. However, the worldwide prevalence of child malnutrition, including stunting, underweight, and wasting, remains persistently high and is concentrated in low- and middle-income countries (LMICs). In 2020, stunting affected 32.3% and 32.5% of children aged 0–59 months in Eastern/Southern and West/Central Africa, respectively. The prevalence of stunting in East Asia (13.5%) and Middle/North Africa (15.6%) was far lower, but it was still three to four times greater than that in North America (3.2%)^5^. These findings highlight the importance of accelerating efforts to close this geographical gap in stunting.

According to the United Nations Children’s Fund report “Improving Child Nutrition,” household food insecurity is an important factor for child undernutrition, which is in turn affected by socioeconomic conditions and the national/global context^6^. Food insecurity leads to inadequate dietary intake, which directly affects height and weight and exerts indirect effects by promoting disease occurrence. This was empirically demonstrated by Humphries (2015), who established that children from chronically food-insecure households in Ethiopia, India, Peru, and Vietnam had significantly lower height for age Z-score (HAZ) values than children from households that were consistently food-secure^7^.

Social capital, defined as the resources embedded within social networks^8^, has been demonstrated to positively affect health. Social capital can be analyzed as an individual attribute (e.g., as an individual’s access to social support within a network) or as a collective property collective, (e.g., norms of mutual assistance within a group)^9,10^. Although social capital has long been discussed in the social sciences, the emergence of social capital as a concept of interest in research on health is relatively recent^11^. Several studies have found positive associations between maternal or household social capital and child nutritional status^12–15^. However, it is difficult to reach any definite conclusion because the characteristics of the sample and measures of social capital have varied from study to study, and the results have been mixed^12–15^.

Studies that found positive associations between social capital and child nutritional status suggested that increased food security may be the mechanism, whereby individuals share food resources within their network or gain access to knowledge of where to obtain cheap sources of food. A handful of studies have demonstrated associations between social capital and household hunger or food security. Martin et al. (2004) found a significant association between increased household- and community-level social capital and decreased household experience of hunger in the United States (US)^16^. Similarly, low community- and family-level social capital was associated with higher odds of reporting food insecurity among the elderly in the US^17^. However, most of these studies were conducted in high-income countries (HICs), where food availability is generally relatively high, and therefore, there are abundant sources of support from which food-insecure households can borrow food or receive food assistance. Only one study was conducted in the LMIC setting, and it found a positive association between social capital and food security in six sub-counties of Uganda, three of which participated in a food aid program^18^. Given the significant gap in economic and public health resources between the two settings, it is difficult to generalize the findings from HICs to LMICs^19^. Furthermore, no previous studies examined the mediating role of food security in the effect of social capital on child nutritional status.

The results of prior studies underscore the need to assess the role of food security or food availability in the association between social capital and child nutritional status in LMICs. Although school-age children are old enough to develop their own social networks or to participate in groups while they are still in a growth phase, no studies have evaluated the association between child’s social support and their nutritional status. Unfortunately, the Young Lives (YL) study we utilized does not have enough information to address the comprehensive concept of food security but has information on food availability. Thus, our study aimed to examine 1) whether maternal and child social support is associated with child nutritional status using two indicators: HAZ and body mass index (BMI)-for-age z-score (BAZ); and 2) whether these associations are mediated by food availability.

## Methods

### Study design

For this secondary analysis, data were obtained from the older cohort of the YL study, a longitudinal cohort survey performed in Ethiopia, India, Peru, and Vietnam^20^. The older cohort of the YL study originally comprised 1,000 children in Vietnam and Ethiopia, 1,008 in India, and 714 in Peru aged 8 years when recruited in 2002 (wave 1). Subsequent data were collected at the ages of 12 years in 2006 (wave 2), 15 years in 2009 (wave 3), 19 years in 2013 (wave 4), and finally 22 years in 2016 (wave 5). For our study, wave 1 and wave 2 were used. The YL survey for the older cohort consists of three sets of questionnaires that examine information on households, children, and communities and were administered in interviews with primary caregivers, children, and community key informants, respectively.

The YL survey employed a clustered multistage sampling strategy in each country. At the first stage, 20 sentinel sites were selected in each country by semi-purposive sampling with a slight oversampling of poor sites to serve the main study objective to explore the causes and consequences of childhood poverty^20^. For example, the most food-deficient areas encompassed the sampling universe in Ethiopia. In Peru, the richest 5% of districts were excluded from the sample. However, the final samples represent a wide range of regions, policy contexts, and living conditions^7^. The cohort in India consisted of households only from Andhra Pradesh state, while the cohorts in the other three countries were nationwide. At the second stage, all households with children of the right age within the sites were listed, from which 100 households were randomly selected at each site (Figure S1)^21^.

The response rate was above 90% in all countries. Data were collected from the child’s main caregiver by a standardized, interviewer-administered questionnaire. The data for our analysis were extracted from waves 1 and 2 and limited to the biological mother.

### Study indicators

#### Child anthropometry

Child nutritional status was captured using anthropometric indicators for child height and BMI, which are affected by chronic and acute nutritional status, respectively^22^. In the YL survey, anthropometric indicators were measured by adequately trained staff members who utilized techniques according to the World Health Organization (WHO) guidelines^7,23,24^. Height was measured using stadiometers with standing plates and moveable headboards, which were locally made, and weight was measured by a calibrated digital balance (Soehnle)^7^. The HAZ and BAZ were calculated using the WHO 2007 standard^25^. Extreme z-scores deemed biologically implausible (< − 6 and > 6 for HAZ, and <  − 5 and > 5 for BAZ) were dropped according to the WHO recommendation^26^.

#### Household food availability

Due to the lack of information available for constructing food security, we utilized information on food availability from the household dataset. Food availability was asked differently in waves 1 and 2. In wave 1, respondents were asked whether the household had gotten enough food to eat, while in wave 2, they were asked whether the household had experienced any food shortage in the last 12 months. Responses of “yes” in wave 1 and “no” in wave 2 were coded as 1, indicating that food was available in the household.

#### Social support

Different questions were used to capture social support across waves. In wave 1, only maternal social support was measured, while the survey used in wave 2 measured both maternal and child social support. Information on maternal and child social support was obtained from the household and child surveys, respectively. The Short Social Capital Assessment Tool (SASCAT) was used to measure maternal social capital in wave 1; the SASCAT is a shortened version of the Adapted Social Capital Tool (A-SCAT) that includes three aspects of structural social capital: membership of groups, involvement in citizenship activities, and social support. The SASCAT was validated in Vietnam and Peru^27,28^, and has been widely used in many LMICs^14,29^. We only utilized the social support items for our study. Support received from groups in which the mother participated (support from groups) as well as social support received from different types of individuals (support from individuals) were combined into an index of maternal social support. For support from groups, when respondents answered that they were members of any of seven different kinds of groups (trade unions, community association/co-ops, women’s groups, political groups, religious groups, credit/funeral groups, and sports groups), they were subsequently asked whether they had received any support from that group. For support from individuals, participants were asked whether they had received support from any of nine different types of individuals (e.g., family, neighbors, friends, and so on). A composite score of maternal social support was calculated by averaging the number of “yes” responses, resulting in a score ranging from 0 to 1 that was analyzed as a continuous variable. We also examined maternal support as a categorical variable using a median split (due to high skewness). In wave 2, only financial support, which is one type of tangible (or instrumental) social support, was examined for the mother, while overall social support was examined for the child. Specifically, mothers were asked how many people they could rely on in times of financial need, with seven response options (none, 1, 2, 3 ~ 5, 6 ~ 10, 11 ~ 15, 16 ~ 20, 12 ~ 30, and > 30). Responses were dichotomized into no (none) versus yes (all others). Children were asked whether there was someone who could help in six different types of situations (detailed questions were described in Table S1). The overall level of child support was calculated by averaging positive responses, resulting in a range of 0 ~ 1, which were also dichotomized using a median split. We additionally examined financial support for the child based on the question asking whether the child had someone who could help when they needed pocket money (Table S1).

#### Covariates

Child characteristics included gender (female vs. male), birth order (second, third, and fourth or higher vs. first), and child’s working status (yes vs. no). Information on child working status was derived from a question asking whether the child was involved in any work activities such as farm work or domestic chores. Maternal characteristics included age in 5-year bands (≥ 30 and < 35, ≥ 35 and < 40, and ≥ 40 vs. < 30), education level (completed vs. not completed primary school), and marital status (permanent partner vs. divorced, separated, single, or widowed). Household characteristics included household size (5 or 6, and > 6 vs. ≦4), residential location (rural vs. urban), and wealth quintiles (second, third, fourth and fifth vs. first). The wealth quintile was based on a wealth index ranging from 0 to 1, which was calculated by averaging three variables: housing quality, ownership of consumer durables, and access to services^30^.

### Statistical analyses

First, we reported descriptive statistics on the characteristics of the analytic sample and calculated the average HAZ and BAZ by each covariate (Table S2). We also presented the distribution of maternal and social support and the average HAZ and BAZ by the level of maternal and child social support for each of the four countries (Fig. 2). Next, the associations between the level of maternal or child social support and the child’s HAZ and BAZ at ages 8 and 12 were assessed using multivariable linear regression models. We introduced a community cluster effect ($\varepsilon_{1, } \varepsilon_{2,} , \;{\text{and}}\;\varepsilon_{3}$) to the model using the “cluster” option in the STATA package. The model can be specified as follows:

1 $$HAZ\;or\;BAZ = \alpha_{1} + \gamma \;maternal\;or\,child\;social\;support\; + \emptyset_{1} X_{1} + \varepsilon_{1 } ,\quad {\text{where}}\;\varepsilon_{1} \sim {\text{N}}\;(0,\sigma_{\varepsilon }^{2} )$$

where X_1_ includes control variables except for food availability, and $\varepsilon_{1 }$ is a community-level random effect.

To explore the mediating effect of food availability in the association between social support and child’s HAZ and BAZ, we fit a mediation model. We examined whether maternal or child social support, which showed a significant association with HAZ or BAZ in Eq. (1), is associated with the probability of food availability in the household, using the following reduced-form specification.

2 $$\left( {logit \frac{{p\left( {being\;food-available} \right)}}{{1 - p \left( {being\;food-available} \right)}}} \right) = \alpha_{2} + \beta \;maternal\;or\;child\;social\;support + \emptyset_{2} X_{2} + \varepsilon_{2}$$

where ${\text{X}}_{2}$ includes control variables that showed strong associations with food availability (household size, mother’s education, and wealth level). For mediation to be present, $\beta$ in Eq. (2) needs to be significantly different from 0. Finally, we introduced the food availability variable in Eq. (1).

3 $$HAZ\;or\;BAZ = \alpha_{3} + \gamma^{\prime } maternal\;or\;child\;social\;support + \mathop \sum \limits_{i} \delta_{i} \;Food\;availability_{i} + \emptyset_{3} X_{3} + \varepsilon_{3}$$

**Figure 1:**
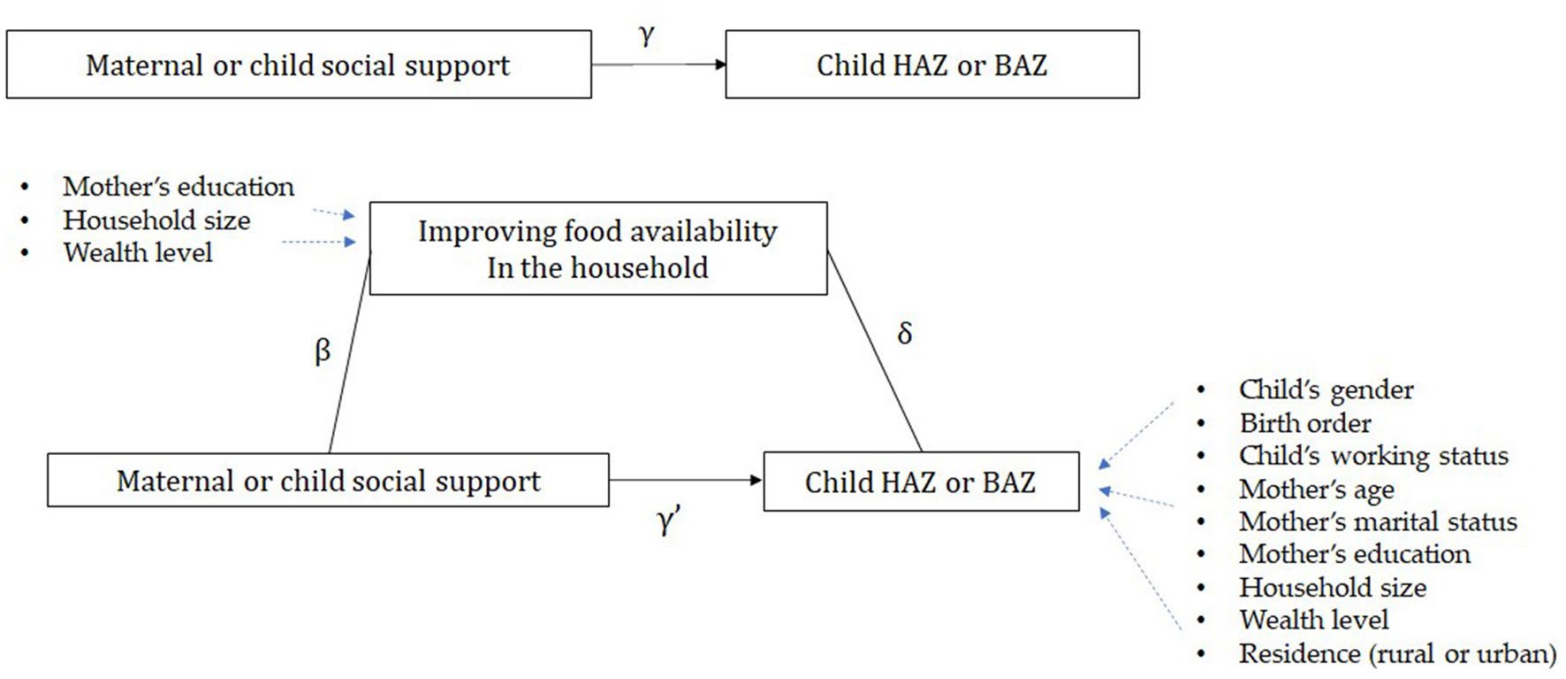
The conceptual framework of the mediation model.

To demonstrate the presence of mediation, δ in (3) needs to be significantly different from 0 and $\gamma^{\prime }$ in (3) must be either 0 or less than $\gamma$ in (1) in absolute value. The relationships described above can be visualized in terms of the mediation model proposed by Baron and Kenny (Fig. 1)^31^.

However, this process does not fit well for non-linear mediators. Further, it is difficult to make a causal interpretation due to potential omitted variable bias. To address these issues, we used the potential outcome framework introduced by Imai et al. (2010), which uses counterfactuals to identify a causal effect and decomposes the total effect of a variable into direct and indirect (i.e., mediational) effects^32,33^. In the model of Imai et al., the total effect is γ in Eq. (1), which corresponds to the total effect of social support on HAZ or BAZ (without the food availability effect). The average direct effect is the mean difference between two counterfactual states of initial conditions, assuming no change in the mediator ($\gamma^{\prime }$ in Eq. (3)), which is a direct effect of social support on HAZ or BAZ after taking into account the mediation (indirect) effect of food availability. Finally, the average causal mediation effects (ACME) is defined as the mean difference in effect between two counterfactual states of a mediator, assuming no change in the initial condition (total effect subtracted by direct effect: $\gamma - \gamma^{\prime }$, which equals the product of $\beta$ in Eq. (2) and δ in Eq. (3)). The mediation analysis was performed using the user-written code -medeff– in STATA 14^34–36^. Analyses were performed separately by wave (age 8 and age 12) and country.

## Results

**Figure 2:**
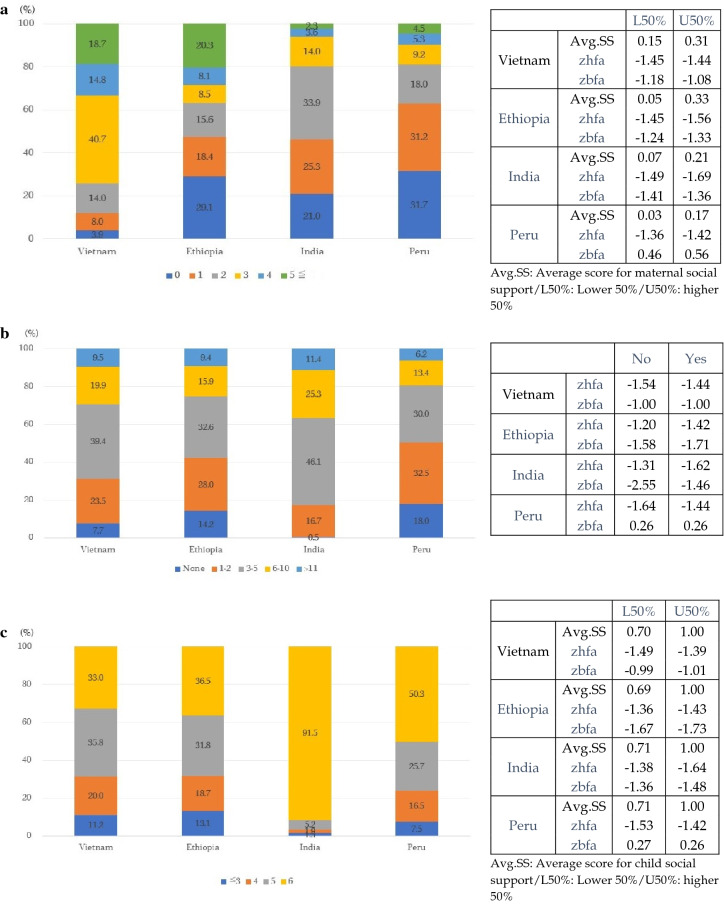
Distribution of social support. (**a**) Distribution of maternal social support in wave 1. (**b**) Distribution of maternal financial support in wave 2. (**c**) Distribution of child social support in wave 2.

Our results were based on complete case analyses of 944, 813, 927, and 634 observations (mother—child dyads) in Vietnam, Ethiopia, India, and Peru respectively (Table S2). The proportion of missing values was less than 10%, except in Ethiopia (with 14.0% missing values in wave 1). However, the composition of the analytic and missing samples was assumed to be tolerably similar, such that the missing data did not lead to bias in our results (Table S3). The pattern of social support significantly differed between settings (Fig. 2). The average score of maternal social support was highest in Vietnam (0.15 in the lower 50% and 0.31 in the upper 50% group) and the lowest in Peru (0.03 in the lower 50% and 0.17 in the upper 50% group). In India, 91.5% of children responded that they had someone to help in all six situations. The relationship between the levels of maternal and child social support and the average HAZ and BAZ was not consistent across the countries.

Table S2 shows the descriptive statistics of the study samples in wave 1 from Vietnam, Ethiopia, India, and Peru, as well as the mean HAZ and BAZ for each category (the characteristics of study samples in wave 2 hardly changed from wave 1 because the YL study is a cohort survey). Generally, children with higher HAZ/BAZ were more likely to be from households with fewer household members, wealthier households, and households in an urban area. The average BAZ for the entire sample was remarkably high in Peru compared to other countries in both waves.

The results for the associations between maternal and child social support and HAZ in waves 1 and 2 without adjusting for food availability were mixed (Table 1). In wave 1, when the child was 8 years old, children of mothers in Vietnam whose overall level of social support belonged to the upper 50% were likely to have *lower* HAZ. No significant associations were found in the other three countries. In wave 2, only Peru showed a positive association between maternal financial support and HAZ. Child financial support was negatively associated with HAZ in Ethiopia, while it was positively associated in Peru. The overall level of child support showed no associations in any countries. As for BAZ, only the level of maternal financial support, operationalized both as a continuous and a binary variable, showed a positive association when a child was 12 years old in India (Table 1).

**Table 1:** Associations between maternal and child support and child z-scores for height and body mass index in waves 1 and 2 in four countries from linear regression adjusted for the community cluster effect.

			Vietnam		Ethiopia		India		Peru	
			b	SE	b	SE	b	SE	b	SE
HAZ										
Wave 1	Overall maternal social support	Continuous	− 0.382	0.309	− 0.252	0.454	− 0.095	0.482	− 0.110	0.828
		Binary (Ref = lower 50%)	–	–	–	–	–	–	–	–
		Upper 50%	− 0.130*	0.061	− 0.055	0.125	− 0.132	0.084	− 0.068	0.091
Wave 2	Maternal financial support	Continuous	− 0.002	0.014	0.025	0.039	0.008	0.054	0.027	0.017
		Binary (Ref = no)	–	–	–	–	–	–	–	–
		Yes	0.022	0.078	− 0.057	0.102	0.064	0.308	0.111*	0.031
	Overall child support	Continuous	− 0.006	0.121	− 0.155	0.126	− 0.276	0.185	0.179	0.119
		Binary (Ref = lower 50%)	–	–	–	–	–	–	–	–
		Upper 50%	− 0.060	0.035	− 0.060	0.060	− 0.123	0.081	0.021	0.047
	Child financial support	Binary (Ref = No)	–	–	–	–	–	–	–	–
		Yes	0.074	0.062	− 0.157*	0.070	0.121	0.140	0.151*	0.065
BAZ										
Wave 1	Overall maternal social support	Continuous	0.367	0.342	− 0.294	0.367	0.549	0.448	0.426	0.437
		Binary (Ref = lower 50%)								
		Upper 50%	0.044	0.066	− 0.124	0.121	0.080	0.077	0.080	0.084
Wave 2	Maternal financial support	Continuous	0.025	0.029	− 0.012	0.039	0.077*	0.036	0.032	0.029
		Binary (Ref = no)								
		Yes	0.115	0.152	− 0.119	0.138	1.217***	0.226	0.003	0.114
	Overall child support	Continuous	0.104	0.187	− 0.166	0.218	− 0.399	0.335	− 0.091	0.181
		Binary (Ref = lower 50%)								
		Upper 50%	0.011	0.088	− 0.014	0.072	− 0.121	0.118	− 0.025	0.067
	Child financial support	Binary (Ref = No)								
		Yes	− 0.058	0.115	− 0.085	0.087	0.127	0.191	0.093	0.093

*b* coefficient from linear regression, *SE* standard error, *HAZ* height for age z-score, *BAZ* body mass index for age z-score (*p < 0.05, **p < 0.01).

Table 2 shows results from logistic regression models examining the association between social support and food availability. As previously described in the Methods section, we limited the analyses only to the social support variables that showed significant associations with HAZ or BAZ (presented in Table 2). The only significant relationship that was observed was a positive association between the level of maternal financial support in Peru and the probability of having enough food in wave 2.

**Table 2:** Associations between maternal and child social support and food security in waves 1 and 2 in four countries from logistic regression adjusted for the community cluster effect.

		Vietnam	Ethiopia	India	Peru
		OR (95% CI)	OR (95% CI)	OR (95% CI)	OR (95% CI)
Maternal support in wave 1	Ref	–			
	Upper 50%	4.64 (0.59–36.56)			
Maternal financial support in wave 2	Ref			*–*	*–*
	Upper 50%			4.43 (0.59–33.08)	1.66 (1.07–2.60)
Child financial support in wave 2	Ref		*–*		*–*
	Upper 50%		0.87 (0.64–1.18)		0.88 (0.48–1.59)

*OR* odds ratio, *CI* confidence interval.

Finally, causal mediation analysis using the method of Imai et al. was performed only in Peru, since it was the only country to show a significant association between maternal financial support and food availability (Table 3). Our model testing the mediating role of food availability in linking maternal financial support to HAZ in Peru showed that the average causal mediation effect of the upper 50% of maternal financial support was less than zero and statistically non-significant at the 95% level, implying that food availability was not a significant mediator of the impact of maternal financial support on HAZ.

**Table 3:** Mediation effect of maternal financial support on HAZ in Peru via securing enough food.

	ACME		ADE		Total effect		% of total effect mediated	
HAZ at wave 2	*having maternal financial support *via* enough food in Peru*							
	− 0.01 (− 0.03, 0.01)		0.12 (− 0.01, 0.24)		0.11 (− 0.02, 0.23)		− 0.08 (− 0.81, 0.32)	

*HAZ* height for age z-score, *ACME* average causal mediation effect, *ADE* average direct effect.

## Discussion

Although extensive efforts have been made to elucidate whether social capital has any benefit on a child’s nutritional status, the results on the effect of maternal social capital have been mixed across studies depending on the type of social capital, child’s age, and geographical location. Furthermore, improved food security has been hypothesized as one of the key mechanisms explaining the positive effect of social capital on child anthropometry, but this hypothesis has never been examined empirically to our knowledge.

Our results do not support a strong or consistent relationship between maternal and child support a child nutritional status. A positive association between maternal and child financial support and HAZ was only found in Peru. However, negative associations were found for overall maternal social support and child financial support in Vietnam and Ethiopia, respectively. In De Silva’s study (2007), the associations between maternal social support and HAZ and weight-for-age z-score of children aged between 6 and 18 months also varied across four LMICs^15^. However, unlike our results, the direction was consistently positive. Although we need to be cautious in the interpretation of negative or non-significant associations between maternal/child social support and child nutritional status, it is postulated that child nutritional status at an earlier age or the nature of social capital as shaped by cultural differences among countries might have affected the results. First, our analyses targeted children aged 8 years old (wave 1) and 12 years old (wave 2), which are older ages than those analyzed by De Silva (2007). Height and weight are more sensitive to feeding status or growth stimulation at a younger age than at later stages of growth. Evidence shows that catch-up growth of preterm infants measured by weight or height mainly occurred from the 10th to 12th months of life^37^. Another study reported that the catch-up growth of malnutrition of institutionalized children who were adopted before the age of 12 months old was much larger than that of children adopted after 12 months old^38^. In light of this, children who suffered from poor nutritional status, such as stunting or low weight at an earlier age, especially before 12 months, would have had greater difficulties catching up to normal children, such that any help from sources of social support may have been inadequate to overcome their growth deficit. Second, since our results are from cross-sectional data, we cannot rule out the possibility of reverse causality (i.e., support is provided to mothers whose children are not growing properly)^15^. Third, there might have been a dark side of social capital—namely, participation in groups might have placed an additional burden on mothers^39^, interfering with their ability to care for their children^15^. A further exploration based on longitudinal data and qualitative studies may be necessary to address this research gap.

Several mechanisms have been proposed to explain the positive effect of social support on child nutritional status. Social support enables mothers to gain knowledge (e.g., how to feed their children for better nutritional status), and give better care (e.g., practicing hygiene habits or breastfeeding longer)^40^. This effect would be more marked in societies where mothers have low levels of education, and therefore, could not have obtained the necessary knowledge through schooling. Emotional support is beneficial for maternal mental health, which can also be linked to improved child growth^41,42^. Martin et al. (2004) provided another theory that social capital is associated with reduced odds of household hunger and food insecurity^16^. Availability and access to food can be enhanced by collectively sharing information and resources. In an LMIC context, sharing seeds and livestock breeds could be good examples. Further, in communities with strong ties, solidarity, and networks, people can share food itself during times of hunger^18^. However, our analyses revealed that child nutritional status was associated only with financial support both for the mother and child, and food availability was not a mediator.

There are several possible explanations for the lack of a mediation effect of food availability. First, the YL study over-sampled households from poor sites, and data from India were collected only in Andhra Pradesh, one of the poorest states. Therefore, food or sources of food (seed or livestock) might not have been sufficient across the community. Even if someone had social support, these sources of support might not have had enough food to share. In Cattel’s (2001) qualitative study, individuals who were part of homogeneous networks of poor households were less likely to receive effective support because other members were not able to provide the required assistance^15,43^. Second, since healthy growth requires a continuous supply of a well-balanced diet, one-off or sporadic support would not be linked to improvements in children’s anthropometric parameters. Cultural specificity of social networks may determine to what extent and how people can give and receive support from each other. Specifically, the type and the nature of social networks might vary across different cultural settings due to different norms^15^. For example, social networks based on religious groups might be stronger in some cultural settings than in others^44,45^. Depending on the cultural context, having someone to rely on in specific circumstances may or may not translate to the availability of long-term and stable support^15^. Questions about the strength of ties with the source of support or the frequency of receiving help from them would help uncover the practical contribution of social support to child nutritional status. Finally, having enough food in the household does not necessarily mean that the household members have consumed enough food. This information also does not reflect the nutritional value or quality of the food.

In light of the above considerations, we are faced with a challenging question: how can a positive association between child anthropometric indicators and financial support to mothers be explained other than through securing enough food? Although further explorations are needed, previous studies on mechanisms linking better economic status and improved nutritional status might provide some clues. For example, improved financial status makes it possible for mothers to access health care services when their children become ill^46^. Furthermore, environmental factors such as improved water, sanitation^47,48^, and cleaner fuel^46,49^ have been found to be associated with improvements in anthropometric indicators. Financial support might have been used to improve these environmental factors rather than to obtain food.

There are several limitations to consider when interpreting the results. First, although the YL study is a cohort survey, we could not exploit the longitudinal design for the analyses because social capital was not uniformly measured across the waves. The cross-sectional analysis limits our ability to draw causal inferences. Second, the levels of maternal and child social support were arbitrarily categorized. We classified the level of maternal and child social support as being in the upper or lower 50% using median cutoff values; this decision was made considering the high skewness and substantially different distributions of data across the countries. However, to reduce the possibility of bias from arbitrary operationalization of the variables, we presented results from models wherein social support was operationalized as a continuous or a binary variable. Third, child nutritional status can be affected not only by food availability, but also by food quality, which can be captured through the concept of food security. Fourth, although the effect of social support might vary depending on the type (e.g., tangible, informational, or emotional support)^50^, the YL study does not have information on the types of social support that mothers received. Fifth, paternal social support and characteristics might also affect food availability or child nutritional status. The lack of detailed information on food security, social support (i.e., measurements of the strength and frequency of support), or paternal characteristics limits the interpretation of our study results. Sixth, although the mother’s health status during ante- and intra-partum periods might affect the child’s nutritional status^51,52^, information on these periods was not gathered in the older cohort survey. This might have confounded our results. Finally, the data for the study are more than 15 years old, which may raise the question of whether the results remain valid in the present context of the study setting. However, the findings of our study still may offer implications to other LMICs currently experiencing similar contexts to those of the study countries in the survey years.

Despite its several limitations, the present study contributes to our understanding of whether interventions to boost maternal or child social support can be a practical approach of improving children’s growth status in a resource-poor setting. Our findings suggest that interventions to strengthen social support in anticipation of a positive effect on improving child nutritional status, especially through facilitating food availability in the household by sharing or supporting, may be unreliable in very poor communities. Furthermore, the observed variability across countries implies that a “one size fits all” approach for enhancing social support may not be appropriate. Future research should seek to examine the effect of social support on child nutritional status in various contexts, including those where food resources are abundant (e.g., communities participating in a food aid program), using the concept of food security.

### Ethical approval and consent to participate

The Young Lives Study provides anonymous, secondary data that are publicly available for scientific use. We downloaded the data without identifiers; thus, the confidentiality of the information given by the participants was guaranteed. Therefore, ethical approval was not required. Participants provided signed informed consent before the study commenced and were assured of confidentiality.

## Supplementary Information

Supplementary Information.

## Data Availability

All methods were carried out in accordance with the relevant guidelines and regulations. Data are available from the UK Data Service website (at https://discover.ukdataservice.ac.uk/series/?sn=2000060). Users are required to register and apply for a password with the UK Data Service and sign a confidentiality agreement before getting access to the data. Users are also asked to inform the UK Data Service and Young Lives of any analysis or publication resulting from their work with the dataset.
