# Supplementary material for: Maternal and child social support and food availability in relation to child growth in four low- and middle-income countries
Source: Sci Rep. 2022 Apr 8;12:5910. doi: 10.1038/s41598-022-09850-1 (PMC8993861; doi:10.1038/s41598-022-09850-1)
Supplement: Supplementary file 1 — Supplementary Information. [file 41598_2022_9850_MOESM1_ESM.docx]

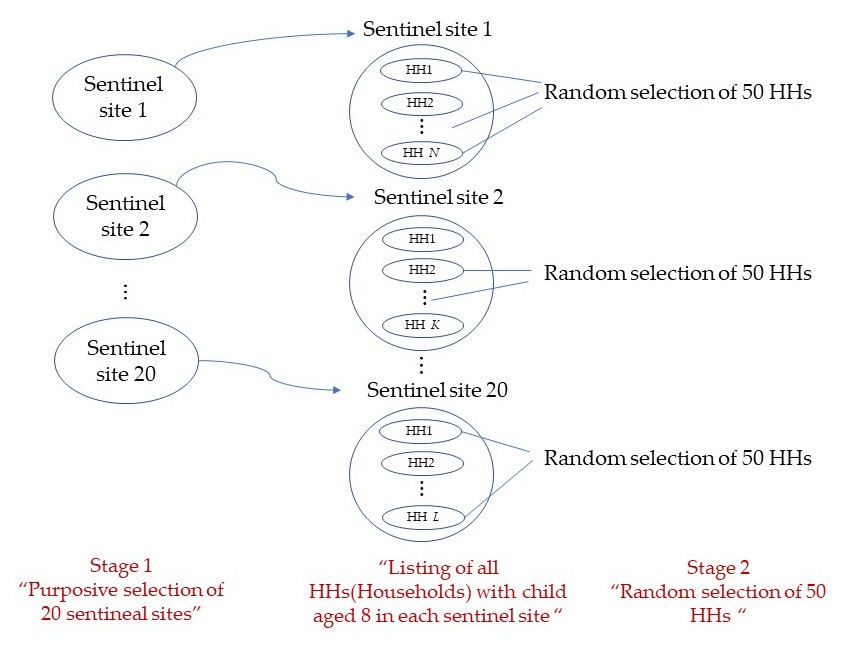


Figure S1. Two-stage sampling process in YL study.

Table S1. Questions for child’s social supports at wave 2.

| Is there someone who could help you~  1. if you were having a problem with your studies at school?  2. if you were worried about something at home?  3. if you were being teased or bullied by another child?  4. if you needed advice about a religious matter?  5. if you needed pocket money?  6. if you needed help getting to school or work? |
| --- |

Table S2. Descriptive statistics of study sample of Vietnam, Ethiopia, India, and Peru (wave 1)

|  | | Vietnam | | | Ethiopia | | | India | | | Peru | | | |
| --- | --- | --- | --- | --- | --- | --- | --- | --- | --- | --- | --- | --- | --- | --- |
|  | | (%) | zhfa | zbfa | (%) | zhfa | zbfa | (%) | zhfa | zbfa | (%) | zhfa | zbfa |  |
| Gender | |  |  |  |  |  |  |  |  |  |  |  |  |  |
| Male | | 49.2 | -1.50 | -1.14 | 52.6 | -1.56 | -1.27 | 49.3 | -1.51 | -1.54 | 53.0 | -1.42 | 0.55 |  |
| Female | | 50.8 | -1.40 | -1.15 | 47.4 | -1.42 | -1.29 | 50.7 | -1.57 | -1.26 | 47.0 | -1.34 | 0.44 |  |
| Birth order | |  |  |  |  |  |  |  |  |  |  |  |  |  |
| 1^st^ | | 36.7 | -1.33 | -1.11 | 16.2 | -1.39 | -1.36 | 27.0 | -1.46 | -1.45 | 30.0 | -1.16 | 0.51 |  |
| 2^nd^ | | 30.4 | -1.34 | -1.12 | 16.6 | -1.59 | -1.14 | 34.3 | -1.49 | -1.37 | 24.9 | -1.29 | 0.40 |  |
| 3^rd^ | | 15.8 | -1.48 | -1.32 | 16.1 | -1.45 | -1.29 | 21.1 | -1.68 | -1.36 | 15.5 | -1.34 | 0.49 |  |
| Higher than 4^th^ | | 17.2 | -1.85 | -1.09 | 51.2 | -1.51 | -1.29 | 17.6 | -1.57 | -1.42 | 29.6 | -1.70 | 0.57 |  |
| Child’s working status | |  |  |  |  |  |  |  |  |  |  |  |  |  |
| No | | 13.7 | -1.42 | -1.12 | 32.6 | -1.55 | -1.29 | 68.5 | -1.55 | -1.41 | 88.4 | -1.33 | 0.52 |  |
| Yes | | 86.3 | -1.45 | -1.15 | 67.4 | -1.47 | -1.27 | 31.5 | -1.51 | -1.38 | 11.6 | -1.75 | 0.31 |  |
| Age of caregiver | |  |  |  |  |  |  |  |  |  |  |  |  |  |
| <30 | | 19.9 | -1.57 | -1.25 | 26.8 | -1.59 | -1.34 | 44.6 | -1.59 | -1.43 | 29.3 | -1.36 | 0.44 |  |
| 30 ≤ & <35 | | 38.5 | -1.42 | -1.13 | 25.0 | -1.48 | -1.19 | 29.4 | -1.48 | -1.36 | 29.1 | -1.35 | 0.54 |  |
| 35 ≤ & <40 | | 23.0 | -1.39 | -1.16 | 25.8 | -1.49 | -1.34 | 17.8 | -1.51 | -1.37 | 19.8 | -1.48 | 0.50 |  |
| 40 ≤ | | 18.6 | -1.43 | -1.04 | 22.4 | -1.40 | -1.24 | 8.2 | -1.50 | -1.45 | 21.8 | -1.36 | 0.52 |  |
| Marital status | |  |  |  |  |  |  |  |  |  |  |  |  |  |
| Permanent partner | | 96.8 | -1.44 | -1.15 | 81.3 | -1.45 | -1.26 | 95.9 | -1.53 | -1.40 | 84.8 | -1.38 | 0.48 |  |
| Divorced or single or widowed | | 3.2 | -1.48 | -1.11 | 18.7 | -1.67 | -1.34 | 4.2 | -1.64 | -1.44 | 15.2 | -1.40 | 0.58 |  |
| Education of caregiver | |  |  |  |  |  |  |  |  |  |  |  |  |  |
| Not completed primary | | 31.4 | -1.70 | -1.04 | 81.3 | -1.49 | -1.38 | 70.1 | -1.65 | -1.44 | 10.4 | -1.83 | 0.42 |  |
| Completed primary | | 68.7 | -1.33 | -1.19 | 18.7 | -1.28 | -0.94 | 30.0 | -1.28 | -1.29 | 89.6 | -1.33 | 0.51 |  |
| Having enough food (wave 1) | |  |  |  |  |  |  |  |  |  |  |  |  |  |
| No | | 1.2 | -1.76 | -1.44 | 22.9 | -1.63 | -1.27 | 2.7 | -1.95 | -0.99 | 18.2 | -1.51 | 0.61 |  |
| Yes | | 98.8 | -1.44 | -1.14 | 77.1 | -1.46 | -1.28 | 97.3 | -1.53 | -1.41 | 81.8 | -1.35 | 0.47 |  |
| Household size | |  |  |  |  |  |  |  |  |  |  |  |  |  |
| 1~4 | | 46.9 | -1.26 | -1.12 | 16.6 | -1.65 | -1.26 | 29.8 | -1.49 | -1.47 | 31.7 | -1.06 | 0.60 |  |
| 5~6 | | 41.2 | -1.54 | -1.21 | 32.8 | -1.48 | -1.30 | 50.1 | -1.49 | -1.36 | 39.4 | -1.52 | 0.38 |  |
| 6≤ | | 11.9 | -1.83 | -1.00 | 50.7 | -1.46 | -1.27 | 20.1 | -1.73 | -1.37 | 29.0 | -1.54 | 0.54 |  |
| Wealth quintile | |  |  |  |  |  |  |  |  |  |  |  |  |  |
| 1^st^ | | 19.8 | -1.76 | -1.02 | 21.8 | -1.70 | -1.26 | 19.9 | -1.65 | -1.51 | 19.8 | -1.83 | 0.35 |  |
| 2^nd^ | | 20.4 | -1.47 | -1.25 | 20.1 | -1.71 | -1.44 | 20.0 | -1.61 | -1.45 | 20.4 | -1.76 | 0.39 |  |
| 3^rd^ | | 19.9 | -1.58 | -1.36 | 19.7 | -1.60 | -1.43 | 20.1 | -1.73 | -1.42 | 20.6 | -1.26 | 0.50 |  |
| 4^th^ | | 20.3 | -1.38 | -1.31 | 19.6 | -1.37 | -1.30 | 20.2 | -1.51 | -1.32 | 19.3 | -1.14 | 0.68 |  |
| 5^th^ | | 19.6 | -1.03 | -0.77 | 18.7 | -1.04 | -0.98 | 19.8 | -1.18 | -1.30 | 19.9 | -0.90 | 0.58 |  |
| Residence place | |  |  |  |  |  |  |  |  |  |  |  |  |  |
| Urban | | 19.4 | -1.08 | -0.77 | 33.4 | -1.20 | -1.02 | 24.5 | -1.22 | -1.29 | 75.1 | -1.24 | 0.57 |  |
| Rural | | 80.7 | -1.53 | -1.23 | 66.6 | -1.64 | -1.44 | 75.5 | -1.64 | -1.44 | 24.9 | -1.82 | 0.29 |  |
| Total | |  |  |  |  |  |  |  |  |  |  |  |  |  |
| 1^st^  wave | Analytic sample | 925 | -1.44 | -1.14 | 760 | -1.49 | -1.27 | 915 | -1.54 | -1.40 | 587 | -1.38 | 0.50 |  |
|  | Original sample | 954 (3.1%) | | | 884 (14.0%) | | | 967 (5.4%) | | | 651 (9.8%) | | |  |
| 2^nd^  wave | Analytic sample | 944 | -1.44 | -1.00 | 813 | -1.39 | -1.69 | 927 | -1.62 | -1.47 | 634 | -1.48 | 0.26 |  |
|  | Original sample | 976 (3.3%) | | | 905 (10.2%) | | | 960 (3.4%) | | | 676 (6.2%) | | |  |

Table S3. Comparison of the sample characteristics of the analytic sample and dropped sample at wave 1 for Ethiopia

|  | Analytic sample (%) | Dropped sample(%) |
| --- | --- | --- |
| Gender |  |  |
| Male | 52.6 | 51.6 |
| Female | 47.4 | 48.4 |
| Birth order |  |  |
| 1^st^ | 16.2 | 20.5 |
| 2^nd^ | 16.6 | 15.6 |
| 3^rd^ | 16.1 | 9.8 |
| Higher than 4^th^ | 51.2 | 54.1 |
| Child’s working status |  |  |
| No | 32.6 | 28.1 |
| Yes | 67.4 | 71.9 |
| Age of caregiver |  |  |
| <30 | 26.8 | 27.4 |
| 30 ≤ & <35 | 25.0 | 21.8 |
| 35 ≤ & <40 | 25.8 | 28.2 |
| 40 ≤ | 22.4 | 22.6 |
| Marital status |  |  |
| Permanent partner | 81.3 | 71.0 |
| Divorced or single or widowed | 18.7 | 29.0 |
| Education of caregiver |  |  |
| Not completed primary | 81.3 | 82.1 |
| Completed primary | 18.7 | 18.0 |
| Having enough food (wave 1) |  |  |
| No | 22.9 | 21.2 |
| Yes | 77.1 | 78.8 |
| Household size |  |  |
| 1~4 | 16.6 | 25.0 |
| 5~6 | 32.8 | 28.2 |
| 6≤ | 50.7 | 46.8 |
| Wealth quintile |  |  |
| 1^st^ | 21.8 | 14.8 |
| 2^nd^ | 20.1 | 23.8 |
| 3^rd^ | 19.7 | 23.0 |
| 4^th^ | 19.6 | 19.7 |
| 5^th^ | 18.7 | 18.9 |
| Residence place |  |  |
| Urban | 33.4 | 29.0 |
| Rural | 66.6 | 71.0 |
